# Direct Comparison of the Efficacy and Safety of Oral Treatments with Oleylphosphocholine (OlPC) and Miltefosine in a Mouse Model of *L. major* Cutaneous Leishmaniasis

**DOI:** 10.1371/journal.pntd.0003144

**Published:** 2014-09-11

**Authors:** Anny Fortin, Diana P. Caridha, Susan Leed, Franklyn Ngundam, Jenell Sena, Tom Bosschaerts, Sandi Parriott, Mark R. Hickman, Thomas H. Hudson, Max Grogl

**Affiliations:** 1 Department of Biochemistry, McGill University, Montreal, Quebec, Canada; 2 Dafra Pharma Research & Development, Turnhout, Belgium; 3 Walter Reed Army Institute of Research, Division of Experimental Therapeutics, Silver Spring, Maryland, United States of America; McGill University, Canada

## Abstract

**Background:**

Cutaneous leishmaniasis (CL) represents a range of skin diseases caused by infection with *Leishmania* parasites and associated with tissue inflammation and skin ulceration. CL is clinically widespread in both the Old and New World but lacks treatments that are well tolerated, effective and inexpensive. Oleylphosphocholine (OlPC) is a new orally bioavailable drug of the alkylphosphocholine family with potent antileishmanial activity against a broad range of *Leishmania* species/strains.

**Methodology/principal findings:**

The potential of OlPC against Old World CL was evaluated in a mouse model of *Leishmania (L.) major* infection in BALB/c mice. Initial dose-response experiments showed that an oral daily dose of 40 mg/kg of OlPC was needed to impact time to cure and lesion sizes. This dose was then used to directly compare the efficacy of OlPC to the efficacy of the antileishmanial drugs miltefosine (40 mg/kg/day), fluconazole (160 mg/kg/day) and amphotericin B (25 mg/kg/day). OlPC, miltefosine and fluconazole were given orally for 21 days while amphotericin B was administered intraperitoneally for 10 days. Ulcer sizes and animal weights were followed up on a weekly basis and parasitemia was determined by means of a real-time *in vivo* imaging system which detects luminescence emitted from luciferase-expressing infecting *L. major* parasites. Amphotericin B and OlPC showed excellent efficacy against *L. major* lesions in terms of reduction of parasitic loads and by inducing complete healing of established lesions. In contrast, treatment with miltefosine did not significantly affect parasitemia and lesion sizes, while fluconazole was completely ineffective at the dose regimen tested.

**Conclusions/Significance:**

Given the data showing the outstanding efficacy and tolerability of OlPC, our results suggest that OlPC is a promising new drug candidate to improve and simplify current clinical management of *L. major* CL.

## Author Summary

Cutaneous leishmaniasis (CL) is a vector-borne parasitic disease transmitted to humans by sandflies and characterized by local ulcerative skin lesions. The disease is linked to poverty in the Middle-East, North and East Africa, South-Central Asia and South America, with 0.7 to 1.2 million new annual cases estimated. In most endemic regions CL treatment relies on injections with pentavalent antimonials, old generation drugs with considerable side effects and long treatment regimens. CL is therefore a highly undertreated disease in need of easy-to-administer, orally bioavailable and well-tolerated agents with broad clinical activity. To date, the only oral drug with acceptable efficacy against leishmaniasis is miltefosine, an alkylphosphocholine with a narrow therapeutic window that limits its use. Given the existing clinical need for CL, we tested the efficacy of oleylphosphocholine (OlPC) in a validated mouse model of Old World (*Leishmania major*) CL. OlPC is a new orally bioavailable drug of the same family as miltefosine with potent and broad leishmanicidal activity. In direct comparison with miltefosine, our results indicate that OlPC induces higher parasite clearance and lesion healing with measurable improved tolerance. These promising observations warrant further research on OlPC as a new drug to improve clinical management of CL.

## Introduction

Leishmaniasis describes a range of visceral and cutaneous disease forms caused by infection with protozoal parasites of the *Leishmania* genus, transmitted to humans by phlebotomine sandflies [1], [2]. Cutaneous leishmaniasis (CL) is characterized by primary localized skin infections that sometimes resolve without treatment, but can also evolve into disseminated, diffuse, or mucocutaneous lesions. In the Old World, CL is caused mainly by *L. major*, *L. tropica* and *L. aethiopica*, whereas in the New World *L. braziliensis*, *L. panamensis*, *L. amazonensis*, *L. guyanensis* and *L. mexicana* are the main causative agents [3]. Based on most recent estimates, about 0.7 to 1.2 million new CL cases occur annually [4]. Treatment of leishmaniasis in most endemic regions relies on multiple intralesional, intramuscular or intravenous injections of pentavalent antimonials, old generation drugs that cause considerable toxicity and have unacceptably long treatment schedules which undermine adherence to therapy and contribute to resistance development [2], [5]. Although in the past decade significant progress was made in the field of antileishmanial drug development with the approval of amphotericin B, paromomycin and miltefosine, considerable disadvantages remain [6]. In particular for CL, treatment regimens are poorly justified and have sub-optimal efficacy. Although local therapy can be used to treat certain forms of CL, procedures such as intralesional injection, cryo- or thermotherapy can be painful and may require local anesthesia [3]. Ointments or creams such as those containing paromomycin (WR279, 396) are more suitable for uncomplicated CL cases, and their efficacy for treatment of New World CL and complicated CL (multiple lesions) is still under study in well controlled clinical trials in Panama and Peru. Whether administered topically or systemically, treatment efficacy against CL is highly variable and depends both on the infecting *Leishmania* strain and on the geographic region [2]. As CL is not a life-threatening disease, treatment recommendation is based on a risk-benefit ratio for every case [2]. In view of the considerable drawbacks of current therapies, in particular the long treatment times and associated side effects, moderate clinical manifestations of CL are likely to be undertreated which increases the chance of patients developing debilitating scars or more severe forms of the disease [3]. Orally bioavailable and well-tolerated agents that are effective against a wide range of clinical CL manifestations are needed, especially against complicated CL. So far the only oral drug with acceptable efficacy against leishmaniasis is miltefosine, an alkylphosphocholine generally used in a long 28-day treatment regimen that associates with dose-limiting gastro-intestinal toxicity [3], [6], [7]. Miltefosine has been tested for CL treatment showing acceptable but variable clinical efficacy [8]. Despite these variable results miltefosine (brand name Impavido) was recently approved by the United States Food and Drug Administration.

Oleylphosphocholine (OlPC) is a new chemical entity belonging to the alkylphosphocholine family showing antileishmanial activity against a broad range of Old and New World *Leishmania* species/strains. While OlPC and miltefosine demonstrate comparable activity *in vitro*, OlPC revealed to be of higher efficacy *in vivo* when tested in a predictive hamster model of visceral leishmaniasis [9]. This study evaluates the value of OlPC for the treatment of Old World CL (OWCL) by testing it in laboratory models of *L. major* infected-mice. These models have undergone internal validation and are reproducible according to industry standards [10].

## Materials and Methods

### Animals

Female BALB/c mice weighing 20–25 grams were purchased from Charles River (Wilmington, MA). The animal protocol was approved by the Walter Reed Army Institute of Research (Silver Spring, MD) institutional animal ethics committee in accordance with national guidelines (protocol number 13-ET-26). Research was conducted in compliance with the Animal Welfare Act, other federal statutes, and regulations that relate to animals and experiments involving animals, and principles stated in the Guide for the Care and Use of Laboratory Animals [11]. The authors abide to the reductionist approach of using animal models in drug development.

### Parasite culture and animal infections

Luciferase-labeled or standard *L. major* promastigotes were cultured in Schneider's medium (Lonza) supplemented with 20% heat-inactivated fetal bovine serum at 25°C. Animals were infected at the base of the tail with 1×10^7^ stationary phase promastigotes. The ulcer areas were measured with a calibrated digital caliper once a week. The average diameter of each tail lesion was calculated as the mean of the horizontal and vertical diameters, and this value was used to calculate the ulcer size area in mm^2^. The following parameters were examined to determine toxicology inequity of the study drugs: cure or distress/death, body weight, general physical and coat appearance.

### Drug products and formulations

Crystalline OlPC was supplied by Dafra Pharma Research & Development (Turnhout, Belgium) while miltefosine was purchased from Panslavia Chemicals LLC and provided by the WRAIR depository (Rockville, USA). Fluconazole was purchased from Sigma-Aldrich (St-Louis, USA) and amphotericin B (Ambisome) from Astellas Pharma US Inc. (Northbrook, USA). Miltefosine and OlPC stock solutions were prepared in 1× PBS and stored at room temperature in the dark for a maximum of 7 days. Fluconazole was dissolved in HECT (in 0.5% (w/v) hydroxyethyl cellulose and 0.2% (0.5% HECT, v/v) Tween-80 in distilled water), then homogenized using a PRO Scientific Inc. Monroe, CT homogenizer. AmBisome was dissolved in double distilled sterile water.

### *In vivo* efficacy, lesion cure model (MLL)

Efficacy was assessed by comparing the suppression of lesion size after 28 days in the drug treated group to that in negative vehicle control as previously described [10]. Percent suppression is defined as {[(LS(-)C)−LS(drug)]/LS(-)C}×100, where LS(-)C = lesion size in negative control and LS(drug) = lesion size in drug group. The threshold for success is a percent suppression which is at least 50% of the positive control amphotericin B [10].

### *In Vivo* Imaging System (IVIS) of luciferase-expressing *L. major*

Luciferin (D-Luciferin potassium salt, Xenogen Corporation, Almeda, CA /Goldbio, St Louis, MO), the luciferase substrate, was intra-peritoneally injected into mice at a concentration of 200 mg/kg 18 minutes before bioluminescence analysis. Mice were anaesthetized with isoflurane (MWI veterinary Supply, Harrisburg, PA) and maintained in the imaging chamber for analysis. Emitted photons were collected by auto acquisition with a charge couple device (CCD) camera (IVIS Imaging System 100 Series) using the medium resolution (medium binning) mode. Analysis was performed after defining a region of interest (ROI) that delimited the surface of the affected area. Total photon emission from each infected tail base area was quantified with Living Image software (Xenogen Corporation, Almeda, CA), and results were expressed in photons/sec.

## Results

### Dose-response efficacy of oral OlPC in the mouse *L. major* lesion cure model

A first set of dose-response experiments was used to assess the capacity of OlPC to cure *L. major* cutaneous lesions in BALB/c mice when given orally. *L. major* promastigotes were injected at the tail base of the mice and local lesions were allowed to develop until they reached optimal lesion size of ∼50 mm^2^. Mice were then grouped (n = 5 per group) based on equivalent average lesion sizes and daily oral treatment with OlPC was initiated. Based on previous data generated in *L. infantum* infected hamsters ^9^, doses of 10, 20, and 40 mg/kg of OlPC were selected to be given for 5 or 10 consecutive days (total doses of 50, 100, 200 and 400 mg/kg) (Table 1). Ulcer sizes were measured from the first treatment day (Day 0) up to Day 28 post treatment start, and compared to those of vehicle treated animals. In this mouse treatment model, dosing of 10 and 20 mg/kg daily for 5 or 10 days had little to no impact on lesion growth, while the dose of 40 mg/kg was able to significantly reduce their sizes. For the 5-day and 10-day regimens, lesion sizes were reduced by 34.0% and 93.5%, respectively (Table 1). The 10-day regimen at 40 mg/kg was independently validated by intraperitoneal (IP) treatment. In this experiment the reduction of lesion sizes was also significant (66.8%, Table 1), although lower than what had been seen with oral treatment. No sign of drug toxicity (as defined in Materials and Methods) was observed in any of the treatment groups. Taken together, these data pointed that an oral daily dose of 40 mg/kg was needed for effective treatment of *L. major* lesions in BALB/c mice, although total disappearance of lesions was not observed with 10 days of treatment.

**Table 1:** Dose-response efficacy of OlPC in the mouse *L. major* lesion cure model (MLL).

Drug	Dose (mg/kg)	Treatment duration (days)	Route	Lesion area on day 28 (mm^2^± SEM)	Suppression compared to control (%)
Vehicle control	-	10	oral	127.7±8.5	-
OlPC	40	10	oral	8.4±3.8	93.5*
OlPC	20	10	oral	112.1±16.3	12.2
OlPC	10	10	oral	159.0±25.4	0.0
OlPC	40	5	oral	84.0±9.7	34.0*
OlPC	20	5	oral	121.0±19.4	5.2
OlPC	10	5	oral	115.1±9.7	9.7
Vehicle control	-	10	i.p.	126.3±18.3	-
OlPC	40	10	i.p.	41.9±9.3	66.8*

* *P*<0.05 compared to vehicle control of the same route.

### Comparative efficacy of oral OlPC to standard treatments in *L. major* infected mice

Building on the previous dataset, the efficacy of OlPC to cure *L. major* induced lesions in BALB/c mice was directly compared to those of the clinically used antileishmanial drugs miltefosine, fluconazole and amphotericin B. For this experiment, mice were infected with luciferase-labeled promastigotes and lesions were allowed to develop until they reached optimal lesion size of ∼50 mm^2^. Mice were then grouped (n = 6 per group) based on equivalent average lesion sizes. To allow direct comparison between treatments, OlPC (40 mg/kg/day), miltefosine (40 mg/kg/day) and fluconazole (160 mg/kg/day [10]) were used orally for 21 days alongside PBS-treated control animals (considering first treatment day as Day 0). As amphotericin B is not orally bioavailable, this drug was administered IP at 25 mg/kg/day for 10 days based on previous experience [10] and served as a positive control. Parasitemia (IVIS), ulcer sizes, and animal weights were followed in each group on a weekly basis.

**Figure 1:**
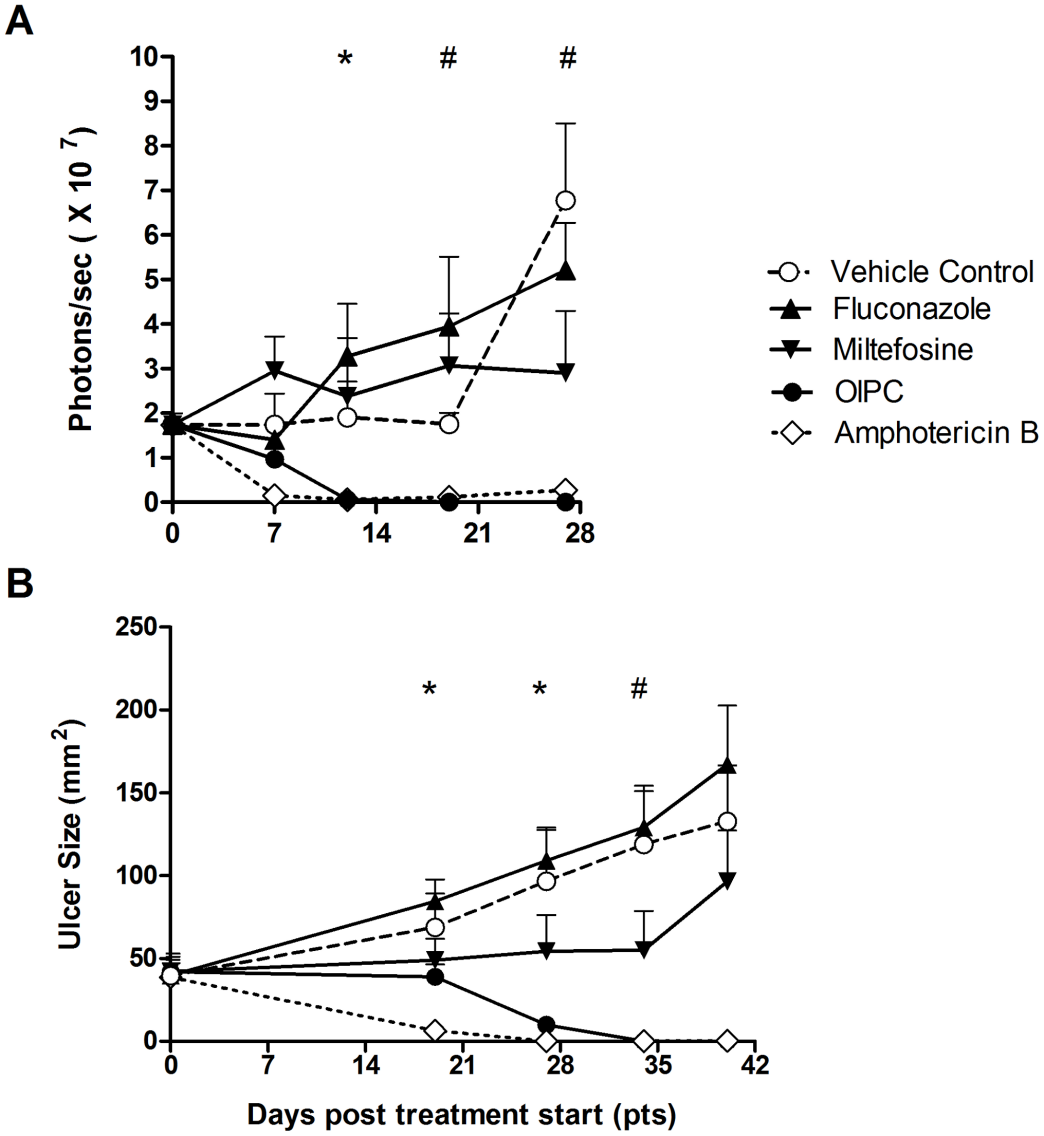
*In vivo* efficacy of oral OlPC against *L. major* in comparison with other antileishmanial drugs. BALB\c mice were infected with luciferase-labeled *L. major* promastigotes at the tail base. When lesions reached ∼50 mm^2^ oral treatment with OlPC (40 mg/kg/day), miltefosine (40 mg/kg/day), fluconazole (160 mg/kg/day), or vehicle control (PBS) was initiated for 21 consecutive days (Day 0–Day 20). Amphotericin B was given intraperitoneally (IP) at 25 mg/kg/day for 10 days (Day 0–Day 9). Parasitemia (A) was measured in every group by *in vivo* imaging (IVIS) on a weekly basis until Day 28 post treatment start and expressed as number of total photons emitted per second. Ulcer sizes (B) were measured weekly using a calibrated digital caliper. Mean ± SEM is shown. * amphotericin B *P*<0.05 compared to vehicle control. # amphotericin B and OlPC *P*<0.05 compared to vehicle control.

As expected, treatment with the reference drug, Amphotericin B, led to a rapid reduction of the parasite loads (visible on Day 7) correlating with lesion size reduction as of Day 19. By Day 27, lesions had healed/cured (defined as 100% re-epithelialization – normal skin) and parasites could not be detected in the mice of this group (Figure 1A, 1B ◊). Although occurring more slowly, response to oral OlPC also led to gradual but complete clearance of parasitemia (seen on Day 12) followed by lesion regression/healing as of Day 27 (Figure 1A, 1B •). In the OlPC-treated group the lesions had completely re-epithelialized/healed by Day 34. In contrast, parasitemia in both miltefosine and fluconazole treated groups never significantly differed from those of the control group, and no lesion regression was observed (Figure 1A, 1B). On Day 34, the control group had an average lesion size of 118.9 ± SEM 32.1 mm^2^, the fluconazole-treated group 129.2 ± SEM 25 mm^2^ and the miltefosine-treated group 55 ± SEM 23.7 mm^2^.

**Figure 2:**
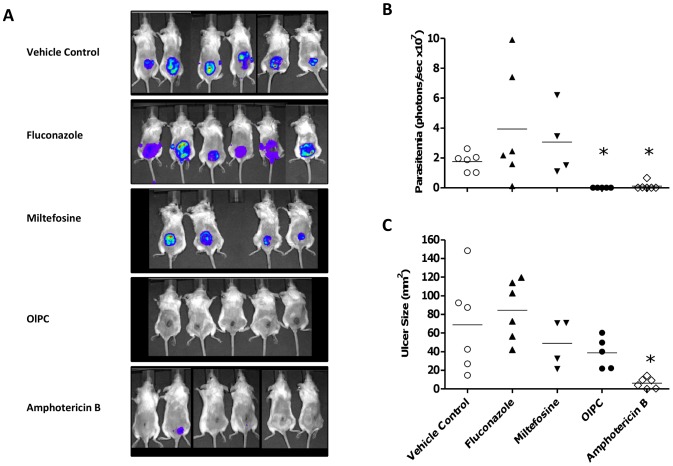
Individual parasitemia and ulcer sizes on Day 19 post treatment start. *L. major* infected BALB/c mice were treated with PBS vehicle control (oral), OlPC (oral, 40 mg/kg/day×21 days), miltefosine (oral, 40 mg/kg/day×21 days), fluconazole (oral, 160 mg/kg/day×21 days) and amphotericin B (IP, 25 mg/kg/day×10 days). Parasitemia (A and B) were measured on Day 19 using *in vivo* imaging technology (IVIS) and ulcer sizes were measured using a calibrated digital caliper (C). Means are shown as horizontal lines (B and C). * *P*<0.05 compared to vehicle control.

A detailed analysis of the Day 19 post treatment start time point is presented on Figure 2, with pictures of the luminescent signal in individual mice (Figure 2A), individual group luminescence values (Figure 2B) and lesion sizes (Figure 2C). The IVIS analysis of OlPC-treated mice clearly shows that OlPC is highly effective at clearing parasites at the lesion site despite the fact that the lesions have not yet started to regress in size. Although the OlPC- and miltefosine- treated groups show similar average lesion sizes at this time point, the difference in the activities of both drugs is nevertheless unambiguous.

Mice remaining beyond Day 34 were closely monitored until the lesion grew to a size >200 mm^2^ or until recrudescence of the ulcers, which were considered as clinical end points. Mice of the control group were euthanized as of Day 35 due to excessive lesion sizes together with the fluconazole-treated mice, indicating that fluconazole was ineffective at the selected dose regimen. Miltefosine treatment appeared to slow down the progression of the ulcers up to Day 34 (Figure 1B) suggesting partial efficacy at 40 mg/kg/day×21 days. However the lesions showed enlargement as of Day 40 (end point 96.4 ± SEM 30.8 mm^2^). In contrast, both OlPC and amphotericin B-treated animals remained lesion free up to Day 54. On day 74, which was the final time point evaluated, both the amphotericin B and OlPC mice had relapsed, showing average ulcer sizes of 31.3 ± SEM 14.9 mm^2^ and 28.4 ± SEM 14.6 mm^2^, respectively (not shown). In conclusion, although treatment with oral OlPC had a slower action than IP amphotericin B, the overall capacity of both drugs to clear the infection in the studied model appeared to be similar and far superior to the one of oral miltefosine at equivalent dose.

### Comparative toxicity assessment and tolerance to treatments

A moderate weight loss was observed in all treatment groups during the 35-day follow-up period, which generally correlated well with disease progression in terms of lesion size. The average weight loss reached a maximum of 8.9% in the vehicle control group and 13.4% in the fluconazole-treated group on Day 34 (Table 2). The miltefosine-treated mice experienced higher weight loss during the treatment period, with an average of 20.6% weight loss on Day 13 (i.e. mid-treatment), indicating potential drug safety issues at the dose used. Amphotericin B- and OlPC- treated mice both experienced a ∼7% weight loss during treatment (peaking on Day 10 and Day 13, respectively), followed by overall weight gain compared to baseline by Day 34 post treatment start.

**Table 2:** Weight variations in treatment groups during comparative efficacy study of OlPC in *L. major* infected BALB/c mice.

Day	Vehicle control	%^1^	fluconazole	%	miltefosine	%	OlPC	%	amphoB	%
Baseline	22.8±0.4		23.1±0.5		24.2±0.7		22.6±0.3		22.7±0.3	
	n = 6		n = 6		n = 6		n = 6		n = 6	
4	22.3±0.3	−2.2	22.4±0.3	−3.0	22.0±0.6	−9.3	21.5±0.5	−4.9	22.3±0.3	−1.8
	n = 6		n = 6		n = 6		n = 6		n = 6	
10	21.2±0.3	−7.1	21.2±0.3	−8.2	20.6±0.2	−14.9	21.7±0.4	−4.0	21.1±0.5	−7.1
	n = 6		n = 6		n = 6		n = 6		n = 6	
13	21.1±0.4	−7.5	20.8±0.4	−10.0	19.3±0.8	−20.6	21.0±0.8	−6.9	23.1±0.2	+2.0
	n = 6		n = 6		n = 4		n = 5		n = 6	
27	21.3±0.5	−6.4	20.7±0.4	−10.4	22.0±1.0	−9.1	21.6±0.7	−4.5	23.4±0.4	+2.9
	n = 6		n = 5		n = 3		n = 3		n = 6	
34	20.8±0.7	−8.9	20.0±0.4	−13.4	21.6±0.1	−11.0	23.7±0.6	+5.0	23.0±0.4	+1.5
	n = 5^2^		n = 5		n = 3		n = 3		n = 6	

1 % compared to baseline weight;

2 No weight available for mouse #584 on Day 34.

Animals euthanized or found dead during the first 35-day follow-up period are reported in Table 3. For two of the mice (1 in control group, 1 in OlPC group), any association with drug toxicity is formally excluded. As for the other found dead animals (3 in the miltefosine group, 2 in the OlPC group and 1 in the fluconazole group), the possibility of cumulative toxicity or complications due to daily gavage (or a combination or the two) could not be excluded. Of those, it is interesting to note that the 3 deaths in the miltefosine group occurred earlier (Day 12, 13 and 19) compared to the ones in the OlPC group (Day 22 and 23), and were associated with piloerection, a recognized sign of sickness in mice, and important weight losses (based on last weight measurement before death; Table 3). Gross necropsy in the two found dead animals of the OlPC-treated group (mouse # 581 and #575) revealed no specific pathological findings, and only mouse #575 underwent weight loss during treatment.

**Table 3:** Sacrifices and found dead animals during comparative efficacy of OlPC in *L. major* infected BALB/c mice up to Day 35 post treatment start.

Group	Animal #	Removal Day	Last weight Day	Weight variation %^1^	Removal reason	Comment/ Observations
control	584	35	27	−13.9	Euthanized	Lesion >200 mm^2^
fluconazole	571	19	13	−12.6	Found dead	Normal appearance
miltefosine	566	12	10	−12.2	Found dead	piloerection
	562	13	10	−24.3	Found dead	piloerection
	578	19	13	−23.3	Found dead	piloerection
OlPC	564	11	10	−4.7	Found dead	Throat injury^2^
	581	22	13	+1.3	Found dead	Normal appearance
	575	23	13	−19.0	Found dead	Normal appearance

1 Last weight measurement compared to baseline weight for each mouse.

2 Linked to oral gavage.

In conclusion, both OlPC and amphotericin B showed excellent efficacy against *L. major* lesions in mice by reducing parasitemia and inducing healing of established lesions. In contrast, treatment with miltefosine at the same dosing regimen as OlPC did not significantly affect parasitemia or induce lesion regression, while fluconazole was completely ineffective at the dose tested. OlPC also appeared better tolerated than miltefosine at equivalent dosing regimen.

## Discussion

As human leishmaniasis comprises several clinical syndromes caused by dozens of *Leishmania* species across the globe, it is unlikely that one drug or drug combination will be effective for all clinical forms of the disease [12]. Therefore, the development of new antileishmanial drugs is needed, preferably with a low side effect profile, oral bioavailability, efficacy in a short treatment regimen, and which can be manufactured at low-cost and adapted for use in rural areas [10], [13]. Currently the only orally bioavailable drug for leishmaniasis is miltefosine, an alkylphosphocholine with a narrow therapeutic window mainly due to its gastrointestinal toxicity. Vomiting and/or diarrhea have been reported in every clinical trial performed with miltefosine [8], and although clinical evidence has suggested efficacy against CL, there is a large variation in clinical response and, in particular for OWCL, more data is needed [8], [14], [15]. Its main limitations are treatment compliance and hence potential for selection of drug resistant parasites and teratogenicity (pregnancy must be avoided during treatment and during the following two months). In this study, the two alkylphosphocholines, miltefosine and oleylphosphocholine (OlPC), were compared side by side for efficacy and safety in a mouse model of OWCL. Of note, the daily dose used approximates the human equivalent dose at which miltefosine is generally used in clinical practice against CL, namely 2.5–3.3 mg/kg (corresponding to 30–40 mg/kg/day in mice) [3], but for 21 days instead of the recommended 28 day regimen.

Based on data accumulated so far in two independent rodent models of leishmaniasis, namely *L. infantum* visceral infection in Golden hamsters [9] and *L. major* cutaneous infection here in BALB/c mice, OlPC has greater *in vivo* efficacy and superior safety profile compared to miltefosine when compared at equivalent dose regimen. In addition, although no direct comparison with miltefosine was performed, the clinical efficacy of OlPC against *L. infantum* canine leishmaniasis (CanL) was also demonstrated in naturally infected dogs using a 14-day regimen of 4 mg/kg/day [16], a daily dose exceeding the maximum tolerated dose of miltefosine in that species (recommended miltefosine regimen in dogs: 2 mg/kg for 28 days). Therefore overall, OlPC is better tolerated than miltefosine and has better efficacy (i.e. wider therapeutic window). Since the antileishmanial activity of OlPC and miltefosine is similar *in vitro* [9], the difference in therapeutic windows between the two drugs could result from differences in oral bioavailability, tissue distribution, or in the affinity of the drugs for the parasites. The detailed PK/PD analysis of OlPC vs. miltefosine in animal models is interesting and deserves further attention. These studies will allow efficient translation of the knowledge accumulated in animal models in future clinical studies in humans aiming at comparing the clinical efficacy of OlPC and miltefosine.

The other two comparative drugs used in our study were fluconazole and amphotericin B. Regarding fluconazole, despite the fact that clinical efficacy in CL patients has been reported in the literature against *L. major* CL at 200 mg daily for six weeks in Saudi Arabia [17] and *L. braziliensis* CL with 8 mg/kg (highest dose tested; corresponding to about 100 mg/kg as an equivalent dose for mice) [18], this drug appeared to be ineffective in our study when given at 160 mg/kg×21 days. However it cannot be excluded that fluconazole would still be effective in the context of a longer treatment period in Balb/c mice. As for amphotericin B, this drug came out as the “best” overall (considering speed of recovery, average group weight loss and group mortality). However as this drug was given IP, it most likely had an earlier T_max_ compared to the other drugs given orally. In addition, IP injections require different types of manipulations than oral gavage, which might influence overall group mortality, aside from the fact that the treatment was only for 10 days as opposed to 21 days for the other groups. Nevertheless, despite the differences in the routes of administration, OlPC achieved similar absolute efficacy than amphotericin B in terms of reduction of parasite loads and lesion remission. The fact that OlPC is orally bioavailable represents a huge practical advantage over amphotericin B considering similar efficacy.

Taken together, our study suggests that even though the optimal oral regimen of OlPC against CL still requires further study and optimization, this new alkylphosphocholine opens the possibility of future improvement of CL patient management in terms of having a well-tolerated oral treatment associated with good patient compliance. In this regard the full FDA/EMA-compliant toxicology and safety pharmacology analysis of oleylphophoscholine is being assembled to pave way to further human clinical development in CL/MCL patients. Having a new oral treatment available will also reduce treatment costs, a factor extremely important in remote settings where cold chain distribution and parenteral drug administration remain challenging.
